# Consumers’ Opinions towards Public Health Effects of Online Games: An Empirical Study Based on Social Media Comments in China

**DOI:** 10.3390/ijerph191912793

**Published:** 2022-10-06

**Authors:** Tao Shu, Zhiyi Wang, Huading Jia, Wenjin Zhao, Jixian Zhou, Tao Peng

**Affiliations:** 1 School of Computing and Artificial Intelligence, Southwestern University of Finance and Economics, Chengdu 611130, China; 2 School of Management Science and Engineering, Southwestern University of Finance and Economics, Chengdu 611130, China; 3 Management College, Ocean University of China, Qingdao 266100, China

**Keywords:** online games, public health, consumer opinion, social media comment, natural language processing, NLP

## Abstract

Online game products have fueled the boom in China’s digital economy. Meanwhile, its public health concerns have sparked discussion among consumers on social media. However, past research has seldom studied the public health topics caused by online games from the perspective of consumer opinions. This paper attempts to identify consumers’ opinions on the health impact of online game products through non-structured text and large-size social media comments. Thus, we designed a natural language processing (NLP) framework based on machine learning, which consists of topic mining, multi-label classification, and sentimental analysis. The hierarchical clustering method-based topic mining procedure determines the compatibility of this study and previous research. Every three topics are identified in “Personal Health Effects” and “Social Health Effects”, respectively. Then, the multi-label classification model’s results show that 61.62% of 327,505 comments have opinions about the health effects of online games. Topics “Adolescent Education” and “Commercial Morality” occupy the top two places of consumer attention. More than 31% of comments support two or more topics, and the “Adolescent Education” and “Commercial Morality” combination also have the highest co-occurrence. Finally, consumers expressed different emotional preferences for different topics, with an average of 63% of comments expressing negative emotions related to the health attributes of online games. In general, Chinese consumers are most concerned with adolescent education issues and hold the strongest negative emotion towards the commercial morality problems of enterprises. The significance of research results is that it reminds online game-related enterprises to pay attention to the potential harm to public health while bringing about additional profits through online game products. Furthermore, negative consumer emotions may cause damage to brand image, business reputation, and the sustainable development of the enterprises themselves. It also provides the government supervision departments with an advanced analysis method reference for more effective administration to protect public health and promote the development of the digital economy.

## 1. Introduction

With the development of the Internet and computer technology, online games have gradually penetrated public life, rapidly occupying people’s fragmented time and becoming a form of leisure and entertainment. According to the 49th Statistical Report on China’s Internet Development, the number of online game users in China reached 554 million as of December 2021, 35.61 million more than that in December 2020, accounting for 53.6% of the total Internet users. Further, the total number of applications (APP) monitored in the Chinese market was 2.52 million, while the number of game APPs continued to lead with 709,000, accounting for 28.2% of all APPs [1]. According to the 2021 China game industry report, the sales revenue of China’s online game industry reached CNY 290.3 billion [2]. Under the combined action of technology and market requirements, the online game industry has developed rapidly and promoted the prosperity of China’s digital economy. It can be seen that online games have become the economic pillar industry in today’s Internet era. However, online games have changed the social economy and people’s lives and have some potential public health effects [3,4,5]. The definition of public health refers to the population’s health, with more emphasis on the health problems of the public in various societies [6]. Different social public organizations form the online game consumption market [7,8]. For instance, consumer groups play game products, game enterprises provide goods, and government departments regulate the game industry [9,10,11,12]. Consequently, the online game products set off human health and socially affect a wide range of populations, which conforms to the connotation of public health: all the problems related to human health can be understood as public health problems [13].

In 1989 the World Health Organization (WHO) redefined health as a state of complete physical, mental, social, and ethical well-being and not merely the absence of disease or infirmity. Therefore, a large number of papers investigating the impact of online games have focused on people’s health by studying the potential harmful effects, involving mental disorders [14,15,16,17] and addiction [3,18,19,20,21], with physical injuries such as obesity, back and neck pain, orthopedic/joint muscle disorders, vision problems, hearing problems, and physical inactivity [22,23]. In addition, regarding social adaptability and ethical health aspects, some research shows that chronic engagement in online gaming is associated with poor social skills, learning difficulties, aggression, ignored family responsibilities, job dereliction, and gambling [5,12,24,25,26,27,28]. Based on the existing state of research, these viewpoints highlight the possible public health problems that can result from online games. A recommendation will be made as to how preventive aspects can be adequately considered in government regulatory policies [11,29,30] and business marketing strategies [31,32,33], which aim to create a healthy and orderly online game industry market environment in China [8,34].

Notwithstanding the endeavors described, little research focuses on Chinese consumers’ opinions on public health effects data of online games products, which can provide a basis and reference for government policymakers and marketing strategy makers [35]. For instance, Cavero Esponera et al. conducted telephonic interviews to gather public opinion on Spain’s food policies to combat obesity [36]. The study found that different groups showed different levels of support for different food policies. Austhof et al. used a global warming audience segmentation tool, a secondary analysis of the consumer panel’s opinion about a public health online questionnaire survey in the online crowdsourcing market [37]. Devlin et al. studied parents’ opinions towards the water-only drink policy at junior triathlon events [38]. Research has found that parents view policy positively, which warrants further research and policy development to facilitate behavioral change. Xu et al., using an online opt-in panel in China, collected survey responses from a total of 1089 respondents to study the influential factors posited by the spiral of silence theory in shaping people’s perceptions of the overall public opinion towards food safety issues and their willingness to speak out [39].

Nevertheless, with the popularization and development of information technology, social media comments are becoming increasingly important ways for consumers to express their opinions [40,41]. Different from the traditional questionnaires or interview methods to collect opinions, data retrieved from social media can be used to obtain or directly analyze opinions [42] and break the limitations of traditional methods such as quantity, timeliness, period, space span, richness, and objective [43]. Thus, using social media comments to track, analyze, and study available opinions related to public health has become a trend in recent years. Yang et al. [44] used social media comments to explore the causal relationship between public environmental opinions and adaptation strategies. Some research based on social media comments is being applied to analyze public opinion on our responses to the COVID-19 pandemic [42,45,46,47]. The study, data collected from a social media Weibo, examined opinions towards child abuse and made recommendations for the government to improve child protection policies [48]. From the perspective of public opinion about emotion and mental health towards the built environment, we explore geolocated Weibo comments to assess the built environment at different scales and to analyze the correlations between built environment elements and sentiment intensity at different scales [49]. Simultaneously, the digitalization form of comment text on social media relies on computer-assisted analytical techniques. Methods for natural language processing (NLP) from the text aim at automatically analyzing and comprehending human language [50], allowing scholars to easily extract beneficial insights contained in textual datasets while avoiding burdensome computational work [51,52]. Based on social media comments, the diversity of NLP methods is revealed. All six primary NLP methods—text preprocessing, text representation, classification, topic modeling, emotion analysis, and deep learning—are applied in public health studies [44,45,46,47,48,49]. The above studies show that social media comments and NLP methods have given researchers unprecedented opportunities to harvest a wealth of textual data materials and advanced technology for investigating contemporary public health phenomena. However, current research is not yet from a new perspective: adopted social media data to analyze consumers’ opinions towards public health effects of online games studies.

Moreover, some studies further advocate for the effective use of consumers’ opinions on social media comments. It supports government in formulating policies and prompts businesses with adjustment strategies. Accurately reacting to people’s genuine opinions can improve government trust and increase enterprises’ loyalty [53,54]. Given this, our research focuses on consumers’ opinions about online game products bringing about public health effects, using practical and diversified NLP methods, and identifying the semantics of comments on different social media platforms. The study allows us to expand our understanding of online game product-related opinions and points to ways that NLP methodologies can be utilized to improve semantics recognition of social media comments, especially regarding the public health effects of online games. Then, understanding public health topics and emotional tendencies in consumers’ opinions towards online games may have important implications for our social well-being and prevent or mitigate continuing public health effects due to online game production. The findings from this study may help relevant role players in the online game consumer market to target government policies and corporate strategies that can be designed to respond accurately to public opinion. It can promote the online game industry’s sustainable development and virtuous cycle and ensure a win-win situation for public health and economic growth.

The structure of this article is as follows:

- Introduce research materials, data sources and preprocessing, and three NLP tasks that form the implementation steps and models of this study.
- Introduce experimental model setting parameters and evaluation indicators.
- Conduct empirical results and discussions to prove the method’s feasibility and obtain advanced suggestions and means for government management and enterprise operation.

## 2. Materials and Methods

### 2.1. Data Sources and Preprocessing from Social Media Platforms

This study uses Python programming to capture 327,505 public social media comments about online games from 1 January 2020 to 15 March 2022. Data required for research are derived from 6 social media platforms, as shown in Table 1. Among them, Weibo is a social platform that emerged in the early days of the Internet (2009), where users can share brief real-time information. It has more than 340 million active users. Zhihu is the first high-quality Q&A community in China’s Internet. Since 2011, there have been more than 2.5 million active monthly paying users, more than 3 million Q&A contents, and more than 3 billion annual visits. Toutiao is the number one recommendation engine based on data mining, with 260 million monthly active users. Tiktok and Kuaishou are the top two in China’s short video publishing platforms, with 620 million and 380 million daily active users, respectively.

Data preprocessing is the key to obtaining an effective experimental database. In this study, the data preprocessing process is divided into six steps. The first step is to unify the fields. Due to the different data formats of each social platform, we unified the field names of valid content, such as comment content, topic, and comment generation time, in the process of collection. The second step is to clean up irrelevant information. The original comment data contain content irrelevant to this study, which will affect the accuracy of subsequent experiments, such as spam, useless links, punctuation, etc. We use web cleanup technology to filter out invalid comments such as ads and spam links. The third step is the correction of typos. Comments on social media are informal language environments, which are prone to typos and need to be corrected. The fourth step is the substitution of synonyms. Chinese has a lot of synonyms. For the convenience of subsequent experiments, synonyms are replaced according to the original comment data to improve the efficiency of the text mining model. The fifth step is word segmentation. We did this by using Jieba, a Chinese phrase widget in the Python computer programming framework that provides a dictionary of Chinese character prefixes. In addition, users can also customize the dictionary to include words not found in the Jieba dictionary. For example, in this study, words related to online games can be added. The new words added according to the study can ensure higher accuracy in subsequent experiments. The sixth step is stopping words processing. This paper uses the stop word list to prevent unnecessary words in the comment sentences. Stop words refer to some words that are automatically filtered out before or after processing natural language text data in order to save storage space and improve the search efficiency of information retrieval, such as “this” and “that”. The raw data are preprocessed through the above steps to form the online game public opinion corpus required by this study, which contains 327,505 comments as shown in Table 1.

### 2.2. Methods

**Figure 1:**
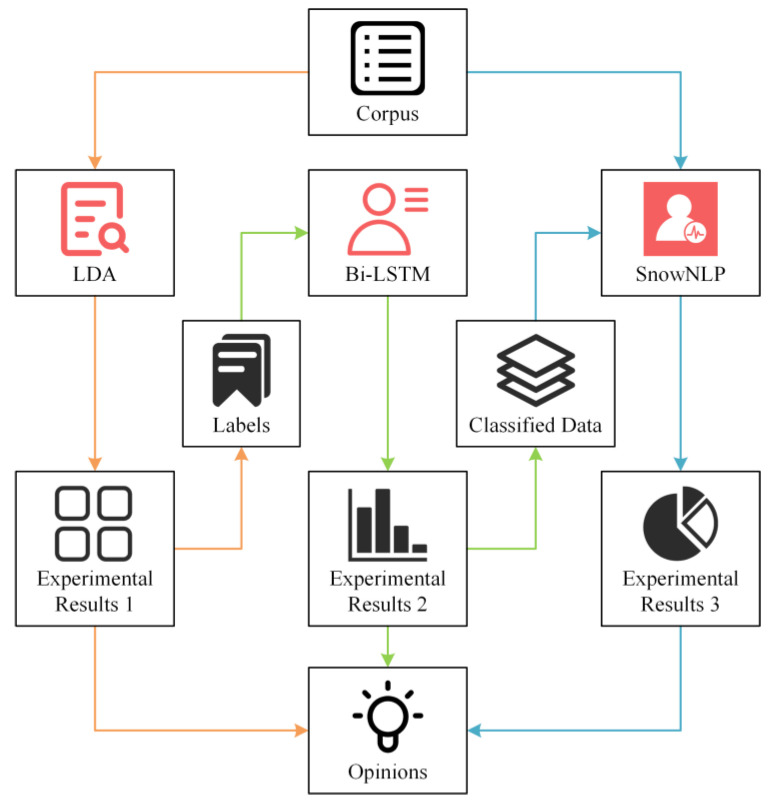
The semantics identification process of consumers’ opinions towards online games.

Natural language processing (NLP) is a field of machine learning to identify important semantic information from large irregular or unstructured databases and extract meaningful knowledge information in the shortest period [55]. Currently, the use of NLP methods to semantically comprehend social media comments has become a popular method in management research [56]. To identify and explore consumers’ opinions about online games causing public health effects from social media comments, this study combined three tasks based on NLP methods of topic modeling, multi-label classification, and emotion analysis. The first task is to mine topics from social comments using the Latent Dirichlet Allocation (LDA) model. The second task is to combine topic mining results with a bidirectional long/short-term memory (Bi-LSTM) model for semantic identification. The third task is emotion analysis based on classified data via the SnowNLP model. According to the results of the three tasks, we can analyze public health-related topics about the online game in social media comments, and the emotional intensity of different topics. The specific methods process of this study is shown in Figure 1.

#### 2.2.1. Topic Modeling Based on LDA Model

Topic modeling is a machine learning technique for learning, identifying, and extracting semantic topics from unstructured text corpora, which divides collections of documents into natural groups to understand them separately [57]. LDA is the most widespread and popular method for fitting topic modeling [58,59]. Some studies have used LDA to explore topics in public health related fields. For example, Paul et al. proposed a disease-related topic model that analyzed over 1.5 million public health tweets [60]. The results suggest that more detailed and comprehensive public health information can be obtained from social media. Botsis et al. developed an LDA model to extract adverse event reports from clinical features of vaccines [61], which facilitates automatic review of adverse events. Hao et al. conducted topic modeling and mining for online comments of doctors [62]. The study found that doctors’ experience, skills, and attitudes to patients were the most important factors affecting patients. Han et al. used topic modeling to mine and analyze public opinion related to COVID-19 in China [42]. As these papers prove, the LDA model has been applied to the research of public health management, and it is also suitable for this study to explore the related topics of public health issues caused by online games.

**Figure 2:**
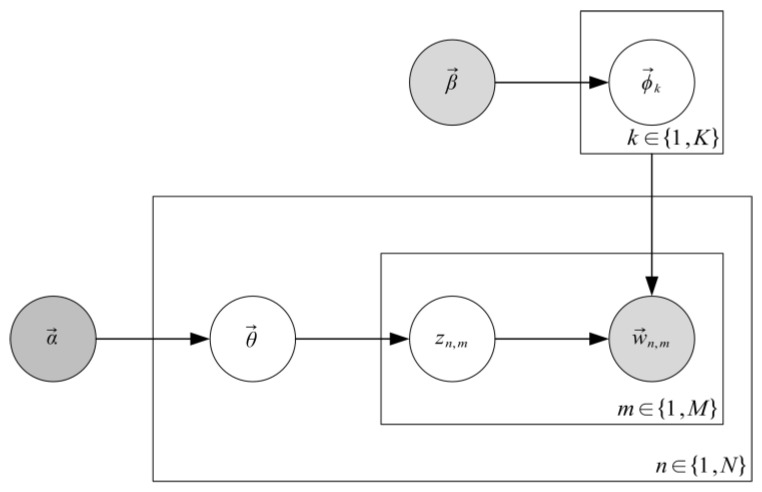
The LDA topic model.

LDA is a three-level Bayesian probability model, proposed by Blei et al. in 2003 [63]. The relationship between topics and words is estimated by hypothesis in the LDA model. A topic consists of multiple words, while a document consists of multiple topics. Figure 2 presents the model structure of LDA, where nodes represent random variables and directed edges represent conditional probability dependence between random variables. In addition, LDA clearly defines the topic distribution $\vec{\theta }$ and the topic word distribution $\vec{\emptyset }$ as a polynomial distribution sampled from the Dirichlet priori with $\vec{\alpha }$ and $\vec{\beta }$ parameters, which makes its generating process more complete and enhances the interpretability and rationality of the modeling process. Assume that corpus $D=\left\{d_{1},d_{2},\cdots ,d_{N}\right\}$, *N* is the contained documents, document $d_{n}$ composed and is expressed as *M* word $d_{n}=w_{n,1},w_{n,2},\cdots ,w_{n,M}$, where each word $w_{n,m}\left(m\in \left\{1,2,\cdots ,M\right\}\right)\;$for the words in the word list, with size *V*. As a generative model for mining *K* implicit topics from corpus, LDA defines the process of generating each word $w_{n,m}$ in document $d_{n}$ as the following two steps: first, sampling an implicit topic $z_{n,m}$ according to the topic distribution ${\vec{\theta }}_{n}$ corresponding to document $d_{n}$; then sample the corresponding word $w_{n,m}$ based on the word distribution ${\vec{\emptyset }}_{z_{n,m}}$ for the implicit topic $z_{n,m}$. Specifically, for the whole corpus *D*, the LDA model has the following generation process:

- For each topic K∈[1,2,⋯,K]:
  i. Sample a word distribution ${\vec{\emptyset }}_{z_{k}}\sim Dir\left(\vec{\beta }\right)$
- For each document $d_{n},n\in \left[1,N\right]$:
  i. Sample a topic distribution ${\vec{\theta }}_{n}\sim Dir\left(\vec{\alpha }\right)$
  ii. For each word in the *N*th document $w_{n,m},m\in \left[1,M\right]$
    a. Sample a topic $z_{n,m}\sim \mathrm{Mult}\left({\vec{\theta }}_{n}\right)$
    b. Sample a word $w_{n,m}\sim \mathrm{Mult}\left({\vec{\emptyset }}_{z_{n,m}}\right)$

#### 2.2.2. Semantic Identification Based on Bi-LSTM Model

Social media comment data identification is a multi-label classification task. Tsoumakas et al. developed a multi-label classification method to automatically identify text [64], as a key technology to process massive text data. The multi-label classification task of text refers to text that is related to multiple labels [65,66]. For example, the comment text “Some online games easily addiction leading to delaying students’ studies, calling for national supervision” contains multiple categories of labels, namely, “online games”, “addiction”, “delay study”, and “supervision”. Moreover, a few multi-label classification models based on deep learning have been proved to improve text identification efficiency, especially in Chinese text [67,68,69,70], among them, including the long short-term memory (LSTM) model [71]. Further, its improved model, such as Bi-LSTM, came to the rescue which effectively leveraged both past and future context information. Through extensive experimentation, researchers have proved that Bi-LSTM is capable of learning way more semantic information than trivial LSTM [72,73]. Therefore, the second task, the multi-label classification Bi-LSTM model, was chosen in this article to identify sematic multiple aspects of public health towards online games in social media comments. In particular, it should be pointed out that the source of classified labels is consumers’ opinion topics that have been obtained, which is the result of the first task.

**Figure 3:**
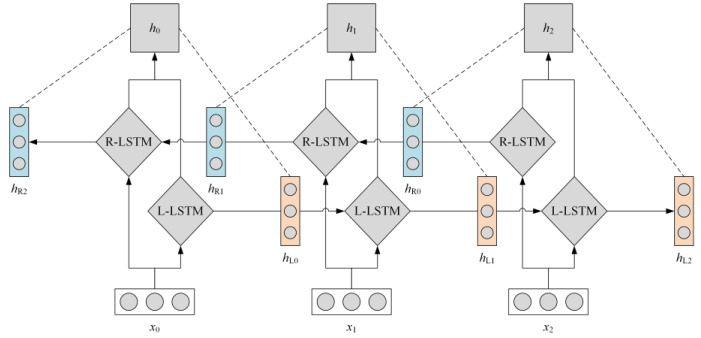
The Bi-LSTM multi-label classification model.

Bi-LSTM, a two-way short and long duration memory network, uses sequential forward and sequential backward outputs to consider both historical and future information at a given moment. The structure diagram of the Bi-LSTM model is shown in Figure 3. As shown in the figure, the input of each time node is passed to the forward LSTM and the reverse LSTM, respectively. Both of them can generate corresponding outputs according to their own state, and these two outputs will be connected together to the output node of Bi-LSTM to form the final output of the model. Moreover, since the timing sequence processing in different directions eventually forms the output together, its training parameters will be optimized to the appropriate value according to the contribution and loss of node output according to the gradient. In fact, the calculation of Bi-LSTM at every moment is very similar to that of ordinary LSTM, assuming that the input sequence of a comment is $w_{1},w_{2},w_{3},\ldots w_{n}—w_{n}$ is the *n* vectorized word. They are in $t_{1},t_{2},t_{3},\ldots t_{n}$, as the input moment of the LSTM model. The calculation process in each LSTM unit can be expressed as:

$$h_{n}=tanh\left(Uw_{n}+Vh_{n-1}\right)$$

where, $h_{n-1}$ represents the hidden layer state of the model at the previous moment of $t_{n}$. U and V represent the weight parameter matrices of the model input layer and hidden layer, respectively. In the final output, the Softmax function will be used to obtain the probability distribution of classification labels, and its calculation formula is as follows:

$$y_{n}=softmax\left(h_{n}\right)=\frac{\exp\left(h_{n\left(i\right)}\right)}{\sum_{j=1}^{T}\exp(h_{n\left(j\right)})}$$

Softmax (.) functions as the activation function. *T* is the number of category labels. $h_{n\left(i\right)}$ represents the *n*th vector component of vector $h_{n}$, whose total vector length is equal to the number of labels *T*.

#### 2.2.3. Emotion Analysis Based on SnowNLP Model

Emotion analysis, the important work of opinion mining, aims to analyze people’s emotional situations towards entities such as topics, services, products, organizations, and their attributes [74,75]. Associated with social media data, automatic emotion analysis of the data is helpful to grasp public opinions in order to make corresponding decisions [76,77].

Bayesian classifiers SnowNLP (Available online: https://github.com/isnowfy/snownlp, accessed on 20 August 2022), which is the Scikit-learn Python machine learning library, have been extensively used to analyze the emotions of Chinese texts in research on public health. Zhang et al. used the SnowNLP model to analyze the sentiment data of restaurant reviews, and studied the differences in consumer preferences and emotional changes before and after COVID-19 [78]. Moreover, based on Weibo comment data, the SnowNLP model was used to analyze the emotional evolution of epidemy-related topics and visually display them in time and space [79]. Jia et al. collected Weibo public comments on returning to college after the epidemic and analyzed sentiments based on the SnowNLP method [80]. The results showed that students had positive emotions about going back to college. Zhang et al. used a Python script to collect 65,277 valid Weibo comments to assess the environmental risk perception of urban residents [81]. SnowNLP sentiment analysis was used to measure environmental risk perception in 366 cities in China, and the structural proportion characteristics and temporal and spatial differences of environmental risk perception were analyzed.

Based on the application experience of the above research, this paper also uses the SnowNLP model to analyze the consumers’ emotions towards different public health effects of online games and complete the last NLP task. For government and enterprises to grasp the opinion information related to online games’ domains, the emotion analysis task is specially designed, which is based on the results of multi-label classification, that is, to analyze the emotions from different consumer’s opinion regarding recognition results of online games.

## 3. Experimental Settings

### 3.1. Experimental Process Setting

- The experimental process of the first task combines quantitative and qualitative analysis. First, quantitative analysis aims to retrieve topics about online games in the self-built corpus of this study; we use the LDA model to mine feature words and topics from the entire corpus and to generate a topic model. Subsequently, the important reasoning task of the researcher is to analyze the feature words mined by the LDA model to determine the topics [45,82,83,84], thus the qualitative analysis is divided into four steps. In the first step, we invited three experts to participate in the determination of the topics, one doctoral student majoring in business administration and two doctoral students majoring in public health. In the second step, each expert separately analyzed the feature words corresponding to each topic and labeled the topic according to prior knowledge. In the third step, the three experts discussed the topics marked separately and decided on the number and name of the topics by voting. In the last step, we invited two professors of related disciplines to review the topics and finally determine the results. Moreover, in order to eliminate the correlations and retain the independence of determinate topics, hierarchical cluster analysis was adopted to build a tree diagram for topic grouping. Hierarchical cluster analysis was used to convert a set of possibly correlated observations into a set of linearly uncorrelated components [44]. Based on the results of clustering, the topics were classified into different domains in consumers’ opinions on the effects of online games.
- The experimental process of the second task is divided into three steps. In the first step, we manually annotate 10% of the corpus data by referring to the topics and feature words determined in the first task to form a data set with marked topics. In the second step, labeled data sets will be randomly divided into training, validation, and test data subsets in a ratio of 8:1:1 for the training and testing of multi-label classification Bi-LSTM model. In the third step, we set the topic mining results of the first task as classification labels and input them into the classification model in order to classify comment data into one or more topics. The classified data set obtained is one of the important results of this study and also the basis of subsequent experiments.
- In the experimental process of the third task, the classified data set obtained in the second task was used as the data base for emotion analysis. Then, the classified data sets were input into the SnowNLP model, and emotion analysis was performed for each topic separately.

### 3.2. Model Parameters Setup

After optimization processes in the experiments, the parameters of the three models applied in this study are finally set as follows:

- The LDA model experimental setup: On the basis of data preprocessing, this article uses a Python third-party module, Scikit-learn, commonly used in machine learning to train LDA subject models, and sets the super parameter alpha value to 0.5 and beta value to 0.1.
- The Bi-LSTM model experimental setup: In this model, the word embedding vector dimension is 256, label vector dimension is 128, hidden layer dimension is 200, optimizer is Adam, batch size is 32, and dropout is 0.4.
- The SnowNLP model experimental setup: On the basis of data preprocessing, this article uses a Python third-party module, Scikit-learn, commonly used in machine learning to train the SnowNLP model, and sets the super parameter alpha value to 0.5 and beta value to 0.1.

## 4. Results and Analysis

### 4.1. The Result and Analysis of First Task

According to the experiment steps of the first task above, we completed the feature word mining and topic determination related to the public health effects of online games in the self-built corpus. The task results are shown in Table 2. The feature word mining results of the LDA model are shown in the fourth column, in which the ten most important feature words for each topic correspond to words of the highest probability in this topic while having a lower probability in other topics. The third column indicates six topics, including “Physical Health” (T1), “Mental Health” (T2), “Individual Virtue” (T3), “Adolescent Education” (T4), “Commercial Morality” (T5), and “Governmental Regulation” (T6), which are determined by the researchers regarding the output of the LDA model. Then, the first column represents the consumer’s opinion domain clustering, including “Personal Health Effects” (D1) and “Social Health Effects” (D2), which come from hierarchical cluster analysis. Furthermore, the advantage of the topic mining task in expanding new topics, as there are two topics reflected in the last column, namely, “Individual Virtue” (T3) and “Commercial Morality” (T5).

According to results above, it is evident that the effects of online game products are related to physical health, mental health, education for young people, and the demand for government supervision. These topics are also common in existing studies. However, further research found that a considerable part of the corpus was distinctly clustered under different topics. It means that some topics paid no attention to, or were less studied before, but occurred in large numbers in the corpus, indicating that consumers have such concerns; therefore, researchers should bring it into the scope of research. First, the undesirable personal behaviors caused by online games also encourage the discussion of consumers, supported by the features words “Unfilial, Lack Ambition, Violence, Gambling, Bullying ...”, etc. It strongly implies a topic related to personal norms of conduct and morality in consumer reviews, named the “Individual Virtue” (T3) topic in this paper. At the same time, consumers also paid additional attention to game enterprises’ behaviors, which is the topic of “Commercial Morality” (T5). The word list strongly supports this topic, “Company Interests, Lure, Devaluation, Game Recharge, Token, Reward, Game Props, Lottery ...”, etc. It indicates that the current mainstream game manufacturers intend to lower the entry threshold and do not seriously verify the user identity to expand the participation population.

In summary, task 1 mined and found a series of public health topics caused by online games in consumer opinions. The total of six topics with the support word list are shown in Table 2. However, the degree of consumer concern for each topic is different, and the correlation between topics is not the same, which will be revealed in the next task.

### 4.2. The Result and Analysis of Second Task

We obtained semantic identification results by implementing multi-label classification in the second task, as shown in Table 3. According to the sorting of classification results, this study selected the top 30 for discussion. It can be seen from the table that about 38.38% of comments do not involve any topics. It shows that some consumers may only express their emotions about the public health effects of online games through text comments on social media, such as “I have an aversion to online games”. In other words, in social media comments, about 61.62% concern at least one topic, containing 201,821 comments as valid data for our study. In this paper, we defined each concern about the target topics as a consumer opinion. All content in one comment to support any one topic can only be counted once. However, one comment can contain multiple opinions if its content supports different topics. Thus, in the corpus, the total count of opinions from all valid comments is 388,802 divided by valid data number, 201,821, resulting in an average of about 1.93 opinions per comment. It strongly supports the fact that many Chinese consumers not only express their opinion on public health topics regarding online games but also their understanding of the relationship between different topics through social media comments.

Then, according to the results shown in Table 3, “Commercial Morality” (T5) accounts for 11.89%, ranking first among the labels with a sole topic, representing the hottest and most sensitive topic in consumer opinion. Subsequently, among the paired topics, “Adolescent Education” and “Commercial Morality” (T4 + T5) ranked first, accounting for 3.31%. It illustrates consumers’ discussions about the public health effects of online games, which often links the adolescent education effects to the behavior of online game enterprises. Furthermore, the combinatorial semantic expression of more than two topics, such as “Physical Health”, “Adolescent Education”, and “Commercial Morality” (T1 + T4 + T5, 1.15%) or “Physical Health”, “Mental Health”, “Adolescent Education”, and “Governmental Regulation” (T1 + T2 + T4 + T6, 0.69), occupy a significant proportion. It indicates that in China’s online game consumer market, many consumers have profound and comprehensive cognition towards the public health effects of online game products.

Furthermore, according to the statistics of the occurrence of permutation and combination topics, it is found that “Adolescent Education” (T4) co-occurs with other topics most frequently, with more than 10.16% related to it. Furthermore, the combinatorial semantic expression of more than two topics involving topic 4 amounts to 9.61%. It shows that adolescent education is the key factor that is closely related to other topics. Due to this, it can be revealed that many consumers are most concerned that online game products result in adolescent education issues.

**Figure 4:**
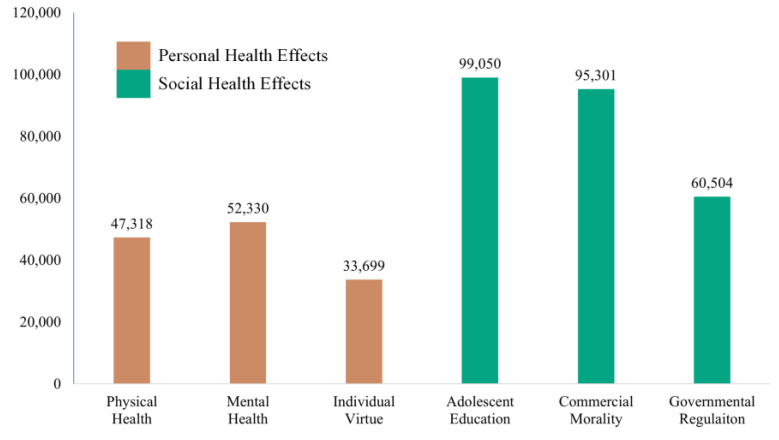
Consumer concern degree of each topic.

Further analysis, in multi-label classification experiments, all labels and their combinations are mapped into the *2^N^* space of the number of topics *N*, thus the results in Table 3 can only represent the number of comments classified under a corresponding individual label. In other words, if a comment involves multiple topics, it will be counted repeatedly. Thus, the total number of comments in the statistical result is greater than the corpus. Ultimately, the consumer concern degree of each topic situation is quantified, as visualized in Figure 4.

Figure 4 shows that the two different colors represent different public health effects caused by online games, among which the consumers’ opinions on the “Personal Health Effects” (D1) domain with 133,347 opinions, and 254,855 opinions are related to the “Social Health Effects” (D2) domain. It reflects the strength of consumers’ concerns towards the public health effects of online games in different domains. Obviously, consumers are more concerned about the social health effects caused by online games, including “Adolescent Education” (T4, 99,050), “Commercial Morality” (T5, 95,301), and “Governmental Regulation” (T6, 60,504); especially topic 4, which is the key factor among all topics, co-occurring most frequently with other topics. At the same time, by referring to the feature words of each topic in the experiment results of task 1, it can be interpreted that consumers’ opinions on online game products are mainly reflected in the fact that they hinder the realization of the social goals of adolescent education. Furthermore, it is rooted in the enterprises’ lack of self-discipline and commercial morality. Thus, consumers have no choice but to appeal for governmental regulation.

Conversely, although online games’ adverse effects on personal health are frequently mentioned in the comments, the level of concern is obviously not as high as the social health effects. It is due to the People’s Republic of China’s online game censorship regime, which lets consumers think that the existing online games have less impact on “Individual Virtue” (T3), with minor comments of 33,699. It shows that under the strict censorship of the Chinese government, game design companies and operating platforms are trying to meet regulatory policy requirements for health content. However, the consumer still perceives the physical and mental effects of online game participants. In particular, the “Mental Health” (T2, 52,330) effect is significantly more significant than “Physical Health” (T1, 47,318).

Finally, in task 2, the completed semantic recognition of consumer comments through the multi-label classification model revealed the honest opinions of consumers that online games affect many aspects of public health, further deconstructed the relationship between various health topics, and quantified the degree of consumer concern for each topic. However, consumer opinions include not only the topic expression of the effects of online games on public health but also the perception and expression of emotions. Therefore, in the next task, it is necessary to analyze each topic’s corresponding emotional intensity further and conduct a multidimensional and fine-granular analysis of consumers’ opinions towards online games’ impacts on public health.

### 4.3. The Result and Analysis of Third Task

**Figure 5:**
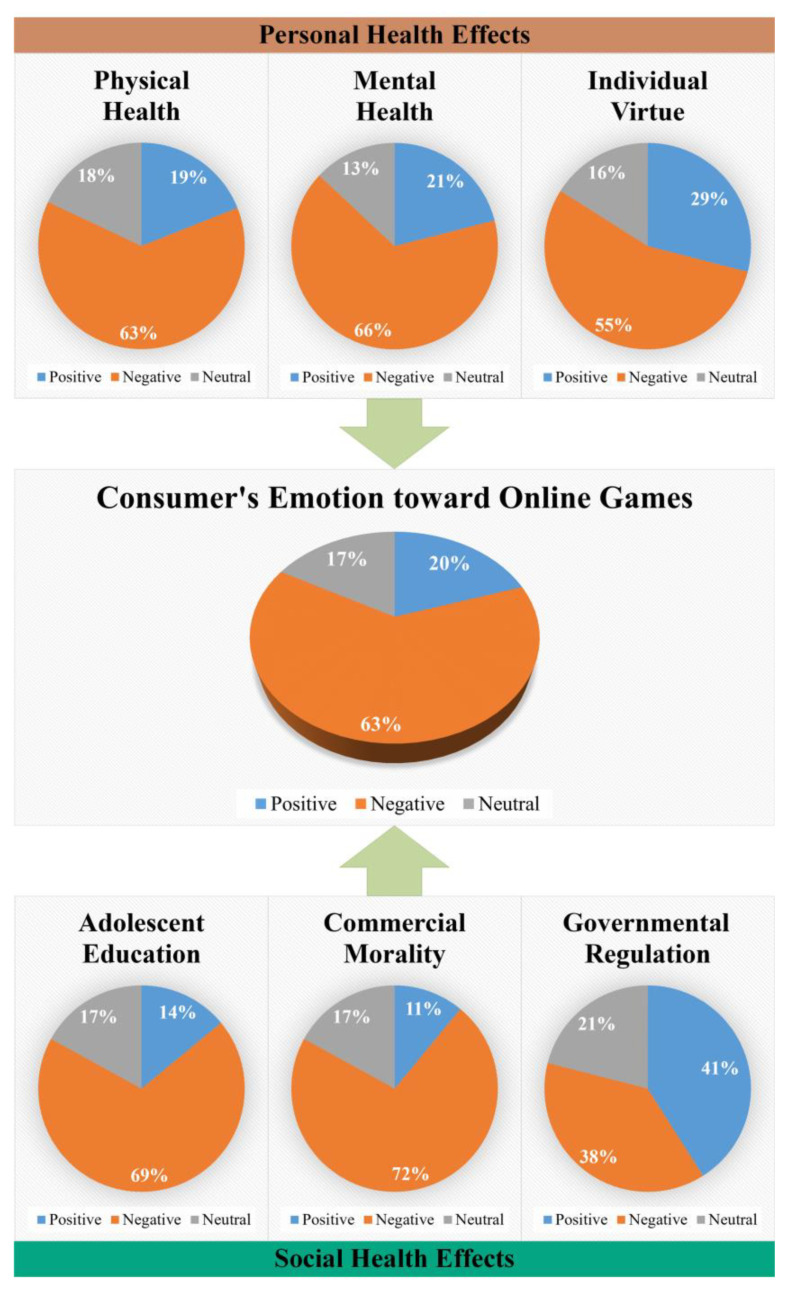
Emotion pie graph of consumers’ opinions on online games.

The result of the third task, each topic emotion classification experiment, identifies consumers’ emotions towards online games causing public health effects. The results are visualized as shown in Figure 5. It can be seen from the figure that the emotion classification pie graph of consumers’ opinions on online games is in the middle. The classification pie graphs of the other six topics surround it, with arrows pointing to it, respectively. It intuitively shows that its classification results are calculated from the results of the six topics. The gray part represents neutral emotions in the seven pie graphs, the blue part represents positive emotions, and the orange represents negative emotions.

As shown in Figure 5, the pie graph of the general emotion of consumers’ opinions on online games shows that negative emotions account for 63%, positive emotions account for 20%, and neutral emotions account for 17%. Thus, the proportion of negative emotions is far more significant than the neutral and positive emotions, indicating that most consumers expressed negative emotions towards related opinions on the public health effects of online games through comments on social media.

Furthermore, in Figure 5, in the domain of personal health effects, the proportion of negative emotions in “Physical Health” (T1), “Mental Health” (T2), and “Individual Virtue” (T3) are 63%, 66%, and 55%, respectively, indicating that consumers have strong negative emotion to the personal health effects caused by online games. Similarly, in the domain of social health effects, the proportion of negative emotions in “Commercial Morality” (T5) accounts for 72%, which is the strongest of the six topics. Conversely, the weakest intensity of negative emotion is “Governmental Regulation” (T6), accounting for 38%. Thus, it can be explained that consumers have strong negative emotions about the public health effects, such as physical health, mental health, adolescent education, and especially commercial morality problems, caused by online games. Yet, from the aspect of government supervision, consumers’ emotional expressions are more positive, holding a supportive trend.

Combined with the results of the first and second tasks in our study, “Commercial Morality” (T5), less studied in the past, not only involves many comments but is also the most potent negative emotion coming from consumers. It indicates that the consumers’ opinions of online games-related enterprises lack self-discipline, commercial morality, and rising popular discontent. Meanwhile, although “Individual Virtue” (T3) is least expressed by consumers in the comments compared to other topics, consumers’ strong negative feelings towards topic 3 indicate that consumers also resent the negative effects of online games on their personal virtues.

Finally, it should be noted that the proportion of negative emotional comments towards “Adolescent Education” (T4) reaches 69%. According to most comments identified by the multi-label classification semantic, “Adolescent Education” (T4) is the most concern of all topics and the most co-occurred topic compared to other topics. It indicates that consumers are worried about the negative impact of online game products on education, especially among adolescents.

The first two tasks mined, identified, and quantified consumers’ opinions on the public health effects caused by online games in social media comments, according to topics. We also have an insight into the relationship between the topics. However, there are differences in consumers’ emotions towards different topics. For example, in “Governmental Regulation” (T6), consumers’ positive emotions outweigh their negative emotions. Thus, the purpose of further emotion analysis for each topic is regarding attitude in this task, whether consumers support or oppose it. The results of this paper may represent the majority of consumers’ cognition towards the health effects of online games.

## 5. Conclusions

### 5.1. Contributions

Based on social media comments, this study uses NLP technology to mine, identify, and reveal consumers’ opinions on the public health effects of online game products. The research results show that six topics related to personal and social health effects domain, respectively, in which the “Individual Virtue” (T3) and “Commercial Morality” (T5) topics are less researched before but objectively exist in consumer social media comments. Additionally, the multi-label classification model quantifies the degree of consumers’ concerns for each topic. It reveals that “Adolescent Education” (T4) is the key factor, which has a large-size number of comments and co-occurs most frequently with other topics. Finally, the emotional intensity analysis was conducted for each topic to obtain different consumers’ attitudes to each topic. It was found that consumers, in addition to “Governmental Regulation” (T6), had negative emotions towards other effects, which for “Commercial Morality” (T5) was the strongest.

This paper summarizes some theoretical contributions based on the above research results.

First, from the new perspective of China’s online game consumption market, this paper innovatively studies the content of consumer opinions on public health effects caused by online game products. It can provide a reference for the government and enterprises, promoting human health, social well-being, and economic development. 

Second, a new comments database based on different social media is established in this study to obtain consumers’ opinions about online games accurately, timely, and objectively. Thus, it provides a new research basis for future researchers to study the public health effects of online games. 

Third, driven by big data, a natural language recognition process for consumers’ opinions on online games was established by using scientific methods. The hierarchical clustering model in this study can practically discover new topics from unstructured text data. It has the ability for precise demonstration and discovery of research topics. The multi-label classification model quantifies the consumer’s degree of concern for each topic and reveals the relationship between topics. Nevertheless, the sentiment analysis task gives emotional intensity to each topic to further deconstruct consumer opinions on online games. The results of these experiments form a complete interpretation of the different dimensions and granularities of the research task for the public.

### 5.2. Practical Implications

Research in this paper has considerable practical significance. Business patterns of most of the current online game companies have the characteristics of low entry barriers and gradually increasing temptation, and have made significant commercial profits. Thus, it is unfeasible to expect the online game industry to reduce the negative impact of this business model on personal and social health through self-discipline. When the harm accumulates to a certain extent, it will inevitably lead to a public backlash. There are currently a lot of highly negative comments by Chinese consumers about the lack of business ethics in the online game industry through social media. Thus, it will bring about more serious social problems and inevitably lead to the intervention of government departments. Suppose the profit of the enterprise is mainly based on this pattern with the intention of attracting consumption and lottery, in that case, the enterprise’s profitability will inevitably decline due to the limitation of government intervention.

It is well known that China is changing from simply pursuing the goal of rapid economic growth to a country that pays more attention to social equality, the health and well-being of the population, especially the youth, and sustainable development [85]. From the perspective of government public management, understanding the relationship between industrial development and public health is conducive to achieving the government’s goals. A series of laws introduced and implemented shows that companies and industries that focus only on short-term benefits and ignore externalities and social responsibilities will be subject to increasingly strict regulations in the foreseeable future [86]. It is actually happening currently, as the Chinese government suddenly tightened restrictions on teenagers’ participation in online games in mid-2021. In 2021, the actual sales revenue of China’s online game market reached RMB 225.538 billion, an increase of RMB 15.862 billion over 2020, with a year-on-year growth of 7.57%. The stock price of Tencent, China’s largest game operator, on NASDAQ fell from about USD 75 in mid-June 2021 to less than USD 40 in mid-September 2022 [87]. While China’s consumer market for online games remains huge and is growing, its growth rate has fallen to its lowest in the past decade, far below the 32.61% growth in 2020 [2].

Moreover, this decline may be fatal to start-ups and small and medium-sized online game companies. Furthermore, since mid-2021, more than 3500 online game enterprises have already been canceled. It can be predicted that most of them are small and medium-sized online game enterprises [88]. Thus, from the perspective of the online game industry-related enterprises, in the face of consumers’ general negative emotions, reminding them of their need to pay attention to the potential harm to consumers while bringing about excess profits through online game products. Otherwise, consumers’ cognition that a large number of various online games lead to public health issues and high negative emotions may damage the overall image of the online game industry, business reputation, and the sustainable development of the enterprise itself. Thus, enterprises should seek their own development strategies, improve their sense of social responsibility, take the initiative to adapt to the new market environment, start from the content design, control the addiction mechanism, explore excellent themes, and seek development opportunities in the new online game field conducive to consumer health and youth education. 

In summary, the research results in this paper can assist the needs of the above two aspects.

## 6. Limitations

Although the current research results have made a contribution, there are still shortcomings. That is, the timeline has not been introduced into the research to obtain the changing trend of consumer opinions. In future research, we will obtain social media comments in different periods to test whether the strategic adjustment of the enterprise is appropriate or whether the government regulatory measures are effective.

Furthermore, although the research object of this paper is online games, its research framework is also expected to be suitable for other industries. China has set new goals for its national development, which is one of the necessary conditions for enterprises to meet the requirements of social health and public well-being requirements. Therefore, limiting this article to the impact of online games on public health is far from adequate, and it needs to be expanded to other industries.

## Figures and Tables

**Table 1:** Data sources and corpus formation.

Social Media Platform	Comments on Online Games
Weibo	34,303
Zhihu	21,789
Toutiao	92,017
Xigua Video	36,547
Tiktok	121,198
Kuaishou	21,651
Total	327,505

**Table 2:** Determined topics and domain clustering.

Opinion Domain Clustering	Topic Code	Topic Name	Top 10 Feature Words Belonging to Each Topic	Appeared in Literate
Personal Health Effects (D1)	T1	Physical Health	Poor Vision, Myopia, Headache, Physical Strength, Nausea, Obesity, Insufficient Sleep, Back Pain, Exercise Less, Physical Weakness	Yes
	T2	Mental Health	Depression, Emotional Stress, Negative Emotions, Nervous Breakdown, Identity Crisis, Avoid Reality, Obsessed, Solitary, Loneliness, Mental Disorders	Yes
	T3	Individual Virtue	Unfilial, Lack Ambition, Violence, Gambling, Porn, Nationalist Sentiment, Patriotic Feelings, Bullying, Family Stability	No
Social Health Effects (D2)	T4	Adolescent Education	Academic Record, Teenagers, Students, Cut Class, Stop Schooling, Ignore Study, Disciplining Child, Rebellious, Lack of Learning Interest, Bad Classroom Discipline	Yes
	T5	Commercial Morality	Company Interests, Lure, Devaluation, Game Recharge, Company Name A *, Token, Reward, Game Props, Lottery, Company Name B **	No
	T6	Governmental Regulation	Industry Governance, Real Name Authentication, Hierarchical Management, Access Restriction, Off Shelf, Shield, Censor Content, Administrative Control, Heavy Fines and Taxes, Green Health Industry	Yes

* The largest online game company. ** Another online game company.

**Table 3:** Semantic identified results of multi-label classification model.

	Proportion (%)	Description
1	38.38%	Only emotion expression, not involving topics.
2	11.89%	T5
3	6.43%	T4
4	5.35%	T6
5	3.13%	T4 + T5
6	2.69%	T2
7	1.94%	T1
8	1.92%	T4 + T6
9	1.89%	T2 + T4
10	1.83%	T1 + T4
11	1.47%	T1 + T5
12	1.42%	T5 + T6
13	1.33%	T3 + T4
14	1.17%	T2 + T5
15	1.15%	T1 + T4 + T5
16	1.04%	T3
17	0.96%	T4 + T5 + T6
18	0.83%	T2 + T4 + T5
19	0.80%	T1 + T4 + T6
20	0.78%	T1 + T2 + T4
21	0.74%	T2 + T3 + T4
22	0.71%	T2 + T4 + T6
23	0.69%	T3 + T4 + T5
24	0.69%	T1 + T4 + T5 + T6
25	0.64%	T3 + T4 + T6
26	0.62%	T3 + T5
27	0.58%	T1 + T2 + T4 + T6
28	0.53%	T1 + T2 + T3 + T4 + T5 + T6
29	0.52%	T2 + T6
30	0.51%	T1 + T2 + T4 + T5
-	-	Those accounting for <0.5% are omitted.

## Data Availability

https://pan.baidu.com/s/1wRfPng-gC2OVjt_4pnrPDA (code: OLph).
